# Using size-selected gold clusters on graphene oxide films to aid cryo-transmission electron tomography alignment

**DOI:** 10.1038/srep09234

**Published:** 2015-03-18

**Authors:** Kenton P. Arkill, Judith M. Mantell, Simon R. Plant, Paul Verkade, Richard E. Palmer

**Affiliations:** 1 School of Biochemistry, University of Bristol, BS8 1TD, UK; 2 Wolfson Bioimaging Facility, University of Bristol, BS8 1TD, UK; 3 Nanoscale Physics Research Laboratory, School of Physics and Astronomy, University of Birmingham, B15 2TT, UK; 4 School of Physiology & Pharmacology, University of Bristol, BS8 1TD, UK

## Abstract

A three-dimensional reconstruction of a nano-scale aqueous object can be achieved by taking a series of transmission electron micrographs tilted at different angles in vitreous ice: cryo-Transmission Electron Tomography. Presented here is a novel method of fine alignment for the tilt series. Size-selected gold clusters of ~2.7 nm (Au_561 ± 14_), ~3.2 nm (Au_923 ± 22_), and ~4.3 nm (Au_2057 ± 45_) in diameter were deposited onto separate graphene oxide films overlaying holes on amorphous carbon grids. After plunge freezing and subsequent transfer to cryo-Transmission Electron Tomography, the resulting tomograms have excellent (de-)focus and alignment properties during automatic acquisition. Fine alignment is accurate when the evenly distributed 3.2 nm gold particles are used as fiducial markers, demonstrated with a reconstruction of a tobacco mosaic virus. Using a graphene oxide film means the fiducial markers are not interfering with the ice bound sample and that automated collection is consistent. The use of pre-deposited size-selected clusters means there is no aggregation and a user defined concentration. The size-selected clusters are mono-dispersed and can be produced in a wide size range including 2–5 nm in diameter. The use of size-selected clusters on a graphene oxide films represents a significant technical advance for 3D cryo-electron microscopy.

Cryo-Transmission Electron Microscopy (cryo-TEM) has become the accepted way of studying proteins and other macromolecular complexes in their native condition. The principle is that the aqueous macromolecules can be immobilized (frozen) and imaged in their native state as long as vitreous ice is formed during the freezing process. Thin-film vitreous ice is the most common way to observe objects in cryo-TEM smaller than a 100 nm. A sample grid, normally a carbon film with holes, has the molecule of interest in an aqueous solution placed on it, after which it is blotted and plunged into liquid ethane (or propane). A thin film layer of glass-like ice is formed in the holes so the molecules or objects of interest smaller than the film thickness can be studied suspended in their original medium. For individual macromolecules it is common to perform single particle analysis, i.e. the collection of many thousands of randomly orientated particles to create a 3D model of the molecule (see for instance, >1300 citations for EMAN, just one software option for reconstruction^1^). However, if the object is larger or has multiple conformations single particle analysis becomes difficult or impossible. Cryo-TEM tomography is a useful tool in these cases, either alone or followed by sub-tomogram averaging^2^,^3^,^4^,^5^. Tomography requires a series of images to be taken of the same object from different projected angles. These images must then be aligned before a reconstruction technique (e.g. weighted back projection) is applied. However, the alignment can be difficult to achieve when the contrast is low, as is usually the case in cryo- TEM, and the location of ‘fixed points’ is difficult. To facilitate this process, dense fiducial markers are introduced to be use as fixed points. Normally these fiducial markers would consist of gold particles suspended within the solution containing the object under investigation. However, achieving a suitable number and distribution of the particles whilst avoiding interaction with the object of interest chemically, or obscuring it in the tomographic reconstruction, can be problematic (e.g. Ref. 6). Graphene Oxide (GO) has been used as a support film in material electron microscopy (e.g. Refs. 7,8) and for single particle analysis^9^. Furthermore graphene-based support films have successfully been used as cryo-tomographic supports^10^, however these were to support a protein rather than to coat over the ice. By using size-selected gold clusters pre-deposited on an ultra-thin support film, we present here a novel way of acquiring cryo-TEM tomograms using Tobacco Mosaic Virus (TMV) as an example.

## Methods

Commercially prepared (Agar Scientific) TEM grids were sourced as a substrate. These arrive covered with a graphene oxide (GO) film supported on top of a perforated carbon film (Quantifoil). Size-selected gold clusters were generated using a magnetron-sputtering, gas-condensation cluster beam source^11^ connected to an inline, lateral time-of-flight mass selector^12^. The nominal mass resolutions achieved using the mass selector were ± 2.4 % for Au_561_ and Au_923_, and ± 2.2 % for Au_2057_, determined using a beam of Ar^+^ ions as a calibration standard. Au_561 ± 14_, Au_923 ± 22_ and Au_2057 ± 45_ clusters from the beam were deposited, sequentially and at the same energy (1.5 keV per cluster), onto separate GO-covered grids in high vacuum. The average coverages were on the order of 10^3^ (for Au_561_ and Au_923_) and 10^2^ (for Au_2057_) particles per square micron. However, it should be noted that the local density of deposited clusters may vary across the substrate, as the cluster beam profile is consistent with a Gaussian shape^11^. Size-selected Au_923_ clusters have previously been deposited onto amorphous carbon^13^ and few-layer graphene^14^ films at energies of 0.5 eV/atom and 5.4 eV/atom, respectively, both below the typical pinning threshold energy for Au clusters on carbon (graphite)^15^,^16^, but they remain monodisperse and sufficiently stable to permit imaging by electron microscopy at room temperature. Subsequently the TEM grids were glow discharged (EDWARDS AUTO 306) to aide wetting. This had no observable effect on the gold clusters.

**Figure 1:**
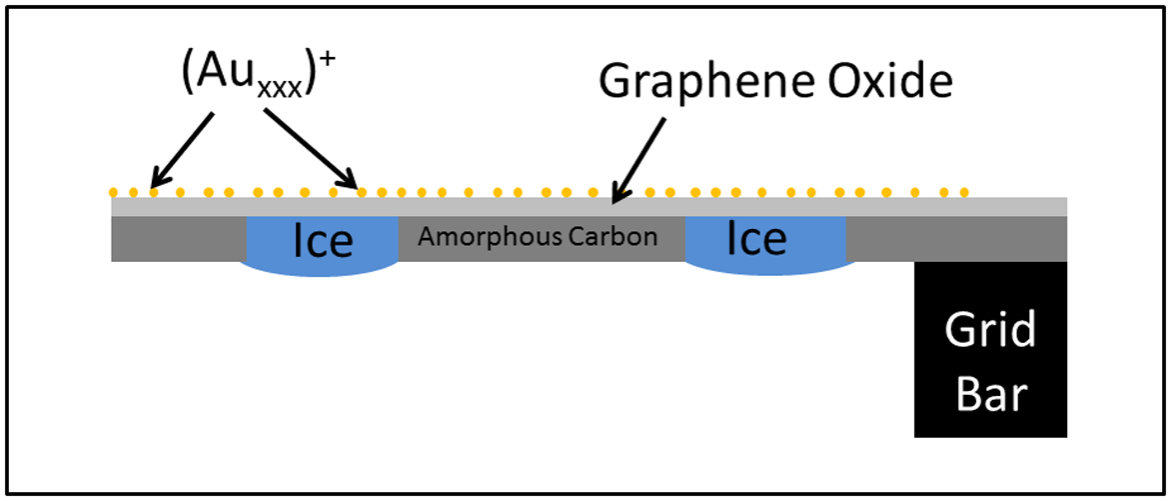
Schematic diagram showing the tomography electron microscopy grid (not to scale). Amorphous carbon with holes is covering a copper mesh electron microscopy grid. A graphene oxide film is supported on top of the amorphous carbon leaving just the graphene oxide over the holes. Au size-selected (by their mass to charge ratio) clusters are deposited on top of the GO film. Vitreous ice containing the sample can be placed on the other side for imaging through the holes.

**Figure 2:**
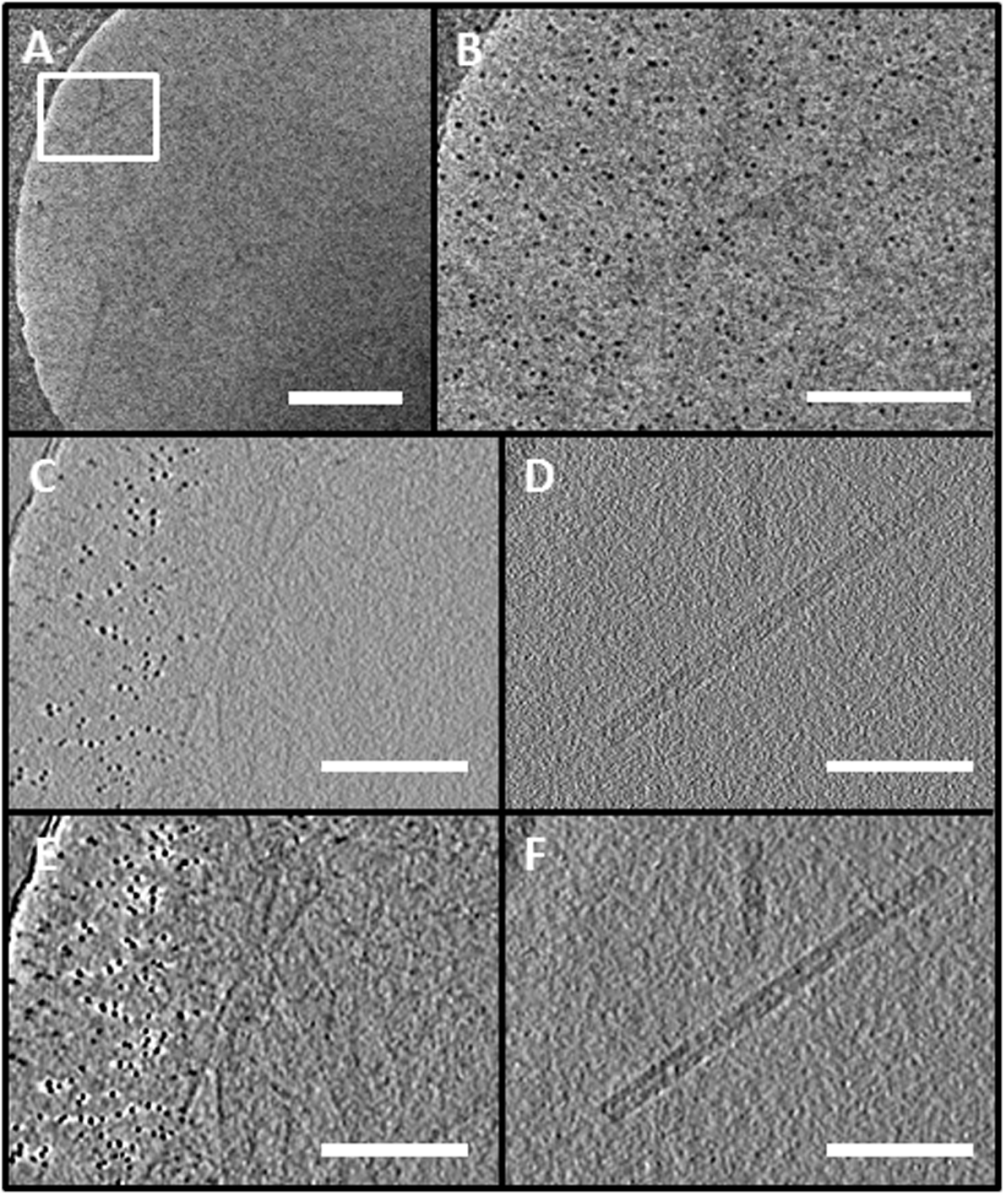
Cryo-tomogram of the TMV using size-selected clusters as fiducial markers supported on graphene oxide (see also the supplemental video). A) Complete field of view of 0 degree tilt from acquired tilt series. B) The boxed area from A) C) Slice from the reconstruction containing size-selected clusters and crinkles in the graphene oxide. Note that not all gold particles are visible in a single slice. The area is approximately that of B). D) Slice from the reconstruction (45 μm above C)) showing the virus. E) and F) are C) and D) after applying a 3D anisotropic diffusion filter. Scale Bar: A) 400 nm, B–F) 100 nm.

Plunge freezing was performed using a VITROBOT mark IV (FEI company) into Liquid N_2_ cooled liquid ethane. 4 μl of TMV in aqueous media was placed on top of the grid and left for 10 s before blotting (1 s) and plunging. This method resulted in a set up where the gold clusters are on one side of the GO layer whereas the vitreous ice is suspended inside the holes of the carbon film on the other side of the GO film (Figure 1). The sample grid was then transferred (without warming) into a Gatan 626 cryo-holder and observed at 200 kV in a Tecnai T20 (FEI company) LaB_6_ TEM fitted with an Eagle 4 k × 4 k camera (FEI) using FEI embedded Low Dose and Xplore 3D Tomography acquisition software. Images in a tilt series were collected at intervals of 2° between ± 50° using a mean of 1 s exposure per micrograph. The off-axis focus condition was used for tracking and worked very efficiently due to the well-spaced and clearly visible gold clusters (Figure 2). The tilt series itself was reconstructed using IMOD^17^ software. The electron flux at 0 degrees was measured as 2.6 e.Å^−2^.s^−1^ (hence 68 e.Å^−2^.s^−1^ total) using the measured current on the phosphorescent screen, i.e. low dose conditions were properly observed.

## Results and Discussion

First, we analyzed whether the small gold particles could be observed and how they behaved in the acquisition and reconstruction of cryo-TEM tomography data. The 3.2 nm gold clusters were clearly visible (Figure 2) at all tilt angles. The reconstruction (Figure 2C-F) clearly shows the TMV above the GO layer and the reconstruction could be improved by standard filtering methods if required. It is noted that curvature in the glass-like ice forms in a similar fashion with and without the GO, i.e. an ellipsoid inside the Quantifoil hole, hence only a subset of the gold clusters is visible in a tomogram slice (Figure 2C). The GO bends away to allow this, though if the ice is on top of the GO with the Quantifoil underneath the curvature is not so great such that the ice is not spread satisfactorily across most of the holes.

**Figure 3:**
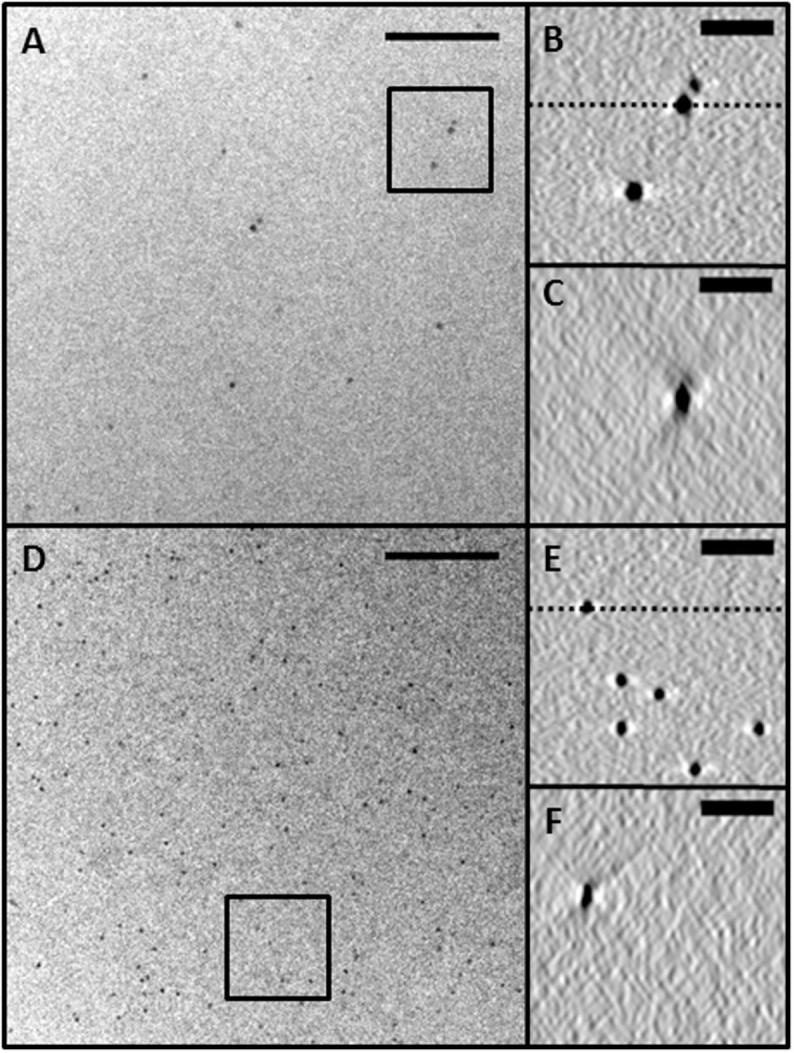
Comparison of drop cast 6 nm diameter Tomosol (Aurion, NL) chemically-synthesized gold fiducial markers and 4.3 nm diameter size-selected Au fiducial markers generated using cluster beam deposition. A) is 6 nm Au markers on GO with vitreous ice on the opposite side of the GO. B) is an XY reconstruction of the boxed area in A). C) is the XZ plan view through the dotted line in B). D), E) and F) are the same as A), B) and C) but for the 4.3 nm Au. Scale Bar: 100 nm in A) and D) and 25 nm in B), C), E) and F).

Next we compared the properties of the deposited gold clusters with those of commonly used suspended gold nanoparticles (Figure 3). For this purpose, a tomogram of only fiducial Au_2057 ± 45_ clusters (diameter ~4.3 nm) supported on a GO film (without an added sample) was reconstructed. This tomogram was then compared with results obtained using the standard Au fiducial marker method of drop casting 6 ± 0.7 nm diameter nanoparticles (Tomosol, Aurion NL) onto the GO. The diameter spread of the standard nanoparticles is the stated maximum from the manufacturer's website. Figure 3 shows the plan view (xy) image and reconstructions obtained using these two different types of markers. The size-selected gold is evenly distributed over the whole imaging area meaning that there are many markers to choose from. The obscured area (including the bright halo) from the fiducial markers in the reconstructions were measured by an intensity profile across the widest point in X and Z (i.e. from orientations such as Figure 3C and F) for >25 Au particles. The Au_2057_ (Figure 3F) affected 12.7 ± 2.1 nm in X and 19.7 ± 3.6 nm in Z. The standard fiducial marker affected 16.7 ± 2.7 nm in X and 27.3 ± 4.4 nm in Z. It was also observed that Au_2057_ with approximately double the atomic density of Au_923_ but with only a slight increase of diameter was easier to consistently observe through thick ice.

The GO layer means tomographic alignment can be achieved by using fiducial markers without having to place them in the solution giving several advantages. The Low Dose focus is normally adjusted in an adjacent quantifoil hole to the area of interest. Using the GO film with Au clusters means that all holes are suitable for focusing and will be focused in the same plane as the area of interest. The user is not dependent on whether good ice has been formed in the hole or, as common practice, have to use the hole edge as a guide. For the exposed area the user can therefore apply an offset to the focus to achieve perfect defocus for objects being imaged. The markers can be numerous and are away from the object of interest in the ice. This helps ensure there is no interaction with the object of interest (even gold exhibits catalytic activity on the nanoscale^18^). The reconstruction has the markers away from the object of interest such that the object is less obscured, and the contribution from the markers could easily be removed from the final tomogram without interfering with the reconstruction. There could be a disadvantage of the markers being only on one surface, compared to throughout the sample but this would be partially negated by the curvature of the support. In the present work, the GO used as the support film, was already commercially available on top of Quantifoil grids at approximately double the cost of a standard Quantifoil grid without the GO. Other graphene derivatives (e.g. fluorinated graphene) or indeed graphene itself could in principle be trialed as a support but we found the GO covers the whole grid evenly and is wet-able. The support itself means there is very little aberration to the main sample, and if required would be removed from the resultant reconstruction as the dimension and location are known. The crinkles which occurred in the GO film are not extensive. The fixed charge density of such supports may have some effect on particles within the first Debye length of circa 1 nm. However, even if there was some alignment this would often not be relevant for the tomographic analysis, which unlike single particle analysis does not require random orientation of particles.

Using size-selected Au clusters deposited from the cluster beam in high vacuum gives some further advantages compared to wet depositing markers. There is no aggregation of particles, which can often obscure points of interest. The microscopist can pre-determine the average spacing and number of the clusters to suit the magnification required to resolve the sample. Cluster size can be determined by the user, can be almost any value, including diameters beween 2 and 5 nm that, though possible, is complex to produce mono-dispersed chemically^19^,^20^,^21^,^22^. We found, in our conditions, that Au_561_ (~2.7 nm diameter) was difficult to track (though it reconstructed well), the Au_923_ (~3.2 nm diameter) was possible to track but the Au_2057_ (~4.3 nm diameter) would be our choice. This, we believe, gave a good balance between density and size. Lastly the clusters are all the same chosen size and so will not be confused, even prior to tomographic imaging, with any other electron dense objects of interest (e.g. immuno-labeled gold).

## Author Contributions

The manuscript was written through contributions of all authors. All authors have given approval to the final version of the manuscript. K.P.A. and R.E.P. lead the project. J.M.M. and K.P.A. took the tomograms and reconstructed the data. S.R.P. carried out cluster beam deposition (including calculations). P.V. contributed to the electron microscopy and scientific discussion.

## Supplementary Material

Supplementary Information Tomographic Reconstruction of TMV
