# Supplementary figures and images for: Using size-selected gold clusters on graphene oxide films to aid cryo-transmission electron tomography alignment
Source: Sci Rep. 2015 Mar 18;5:9234. doi: 10.1038/srep09234 (PMC4363841; doi:10.1038/srep09234)

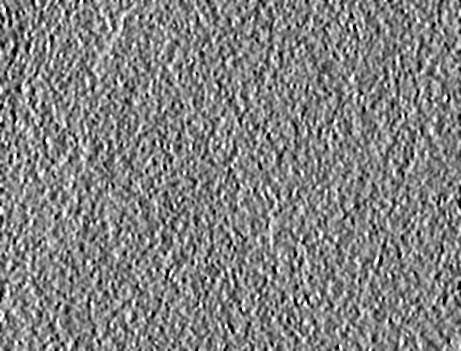

Supplement: Supplementary Information — Tomographic Reconstruction of TMV [file srep09234-s1.gif]
